# Evaluation of the paediatric dose of chloroquine in the treatment of *Plasmodium vivax* malaria

**DOI:** 10.1186/s12936-016-1420-5

**Published:** 2016-07-19

**Authors:** Arletta Añez, Manuel Moscoso, Cecilia Garnica, Carlos Ascaso

**Affiliations:** Departamento de Salud Pública, Universidad de Barcelona, Barcelona, Spain; Laboratorio de Control de Calidad de Medicamentos y Toxicología del Instituto Nacional de Laboratorios en Salud. CONCAMYT-INLASA, La Paz, Bolivia; Departamento de Salud Pública, Universidad de Barcelona. Institut d’ Investicions Biomediques, Augusto Pi i Sunyer, Barcelona, Spain

**Keywords:** Chloroquine, Paediatric doses, Chloroquine in children

## Abstract

**Background:**

Chloroquine (CQ) continues to be the first-line medication used worldwide in the treatment of *Plasmodium vivax* malaria. The dose recommended by the World Health Organization is 25 mg/kg independently of the age of the subject. Nonetheless, the pharmacokinetics and pharmacodynamics of drugs in children are different from those in adults and may influence the drug concentrations in blood and become risk factors for therapeutic failure and/o resistance to CQ.

**Methods:**

This study is a secondary analysis of the data from a clinical trial in which children over 5 years of age were administered 25 mg/kg of CQ, and CQ concentrations in blood were measured at day 7 of follow-up. Models of regression and comparison were used to evaluate and compare the CQ dose taken per kg/body weight, the CQ dose calculated based on body surface area, CQ levels in blood on day 7 and the age of the population.

**Results:**

The younger the study population the greater the difference between the dose per kg/body weight (real dose) and that calculated according to the BSA (theoretical dose). The difference between the two doses was −181.206 mg in the 5–9 years of age group (CI 95 % −195.39; −167.02 mg) and −71.39 mg (CI 95 % −118.61; −23.99 mg) in the 10–14-year-old group. The CQ concentrations in blood on day 7 differed in patients over and under 15 years (p = 0.008). A negative correlation was found between the real and theoretical dose (difference in dose) and the age in years (R2 = 0.529, p = 0.001). A negative correlation was also found between the difference in dose (mg) and CQ concentrations on day 7 (ng/ml) (r = −0.337, p = 0.001). Children under 15 years were found to have a higher rate of therapeutic failure than those over 15 (28 vs 4.2 %, respectively) (Kaplan–Meier p = 0.005).

**Conclusions:**

A CQ dose of 25 mg/kg for the treatment of *P. vivax* malaria may be too low in children as demonstrated by the reduction in CQ concentrations in blood at day 7 of follow-up. This under-dosage is probably associated with the higher rate of therapeutic failure found in children under 15 years (28 vs 4.3 %). These results suggest the need to review the paediatric doses of CQ currently used.

## Background

Vivax malaria continues to be an important health problem worldwide. It is estimated that this parasite caused 13.8 million cases throughout the world in 2015 and contributed to almost half of the cases of malaria reported outside of Africa. In South America, *Plasmodium vivax* causes 70 % of the total number of cases of malaria [1]. Despite the progressive resistance of *Plasmodium falciparum* to chloroquine (CQ) [2, 3], this drug continues to be the medication of choice in the treatment of malaria by *P. vivax* and special cases of malaria [4].

Recognition of the value of CQ occurred late, and this medication was not used until 1946 [5] after having been re-evaluated by the armed forces of the United States for its use against vivax malaria and later for falciparum malaria. The initial studies were carried out in military personnel infected with *P. vivax* at a dose of 1.5 g during four days [6]. The dose recommended by the World Health Organization (WHO) continues to be 25 mg/kg of body weight (BW), divided into three daily doses (1.5 g in patients of 60 kg) independently of the age of the patient [7].

CQ is completely absorbed through the gastrointestinal tract and its maximum concentration is achieved 1–3 h after oral ingestion, with 60 % of the drug binding to plasma proteins [8]. After the ingestion of a single oral dose CQ concentrations in whole blood are approximately tenfold greater than those in plasma. Following absorption, CQ is distributed throughout the body and accumulates in the organs, especially the liver, lungs, spleen and kidneys [9]. The CQ distribution volume (DV) is very large (>200 l/kg), and it rapidly de-alkyates through the cytochrome P450 (CYP) enzymes into pharmacologically active desethylchloroquine (DCQ) and bisdesethylchloroquine. The mean half-life of the drug is from 35 to 40 days after ingestion [10]. The minimum effective concentration (MEC) of the drug in whole blood is 75–150 ng/ml, and, from the most conservative point of view, a CQ + DCQ concentration >100 ng/ml in the presence of parasite growth is considered as resistance [11–13].

Breastfeeding infants and children under many changes along their development in regard to the absorption, distribution, metabolism and excretion of drugs, presenting important pharmacokinetic and pharmacodynamic differences compared to adults [14, 15]. Indeed, these physiological factors may influence the availability of drugs in the organism particularly with respect to body composition as well as extracellular and total body water, fat distribution and muscle mass [16]. Therefore, it has been recommended that drugs should be administered to children according to their body surface area (BSA) [17, 18].

This wide variation in systemic exposure to drug concentrations has a significant effect on the effective drug concentrations achieved within the body. Suboptimal or low CQ concentrations in blood increase the risk of therapeutic failure and the appearance and spread of resistant strains of *Plasmodium* [19–23].

In many studies on the therapeutic efficacy of CQ the rates of therapeutic failure and drug resistance were greater in children than in adults [24–28]. However, no evidence has shown that therapeutic failure in these cases is caused by suboptimal doses of CQ.

On the other hand, the lack of acquired immunity in children affects the spread of resistance to anti-malarial drugs [29], although this seems to be more related to infants [30]. In this respect recent reports have shown that paediatric doses of anti-malarial drugs should be re-evaluated [31, 32].

The aim of this study was to evaluate whether the standard CQ dose of 25 mg/kg achieves the same CQ concentrations in blood of children and adults diagnosed with vivax malaria.

## Methods

This study is a secondary analysis of the data of a clinical trial on the therapeutic response of CQ in infection by *P. vivax* [33]. The analysis was carried out from May to November 2011 in the Amazonic area of Riberalta-Bolivia, which has a medium high level of endemicity [34].

The inclusion criteria were: patients over 5 years of age diagnosed with mono parasitaemia by *P. vivax*, with a parasite density of asexual forms of 250–100,000 parasites/µl. The exclusion criteria were: pregnancy, signs and symptoms of severe malaria, history of allergy to anti-malarial drugs, concomitant severe or chronic diseases such as tuberculosis or HIV/AIDS, and previous treatment with other anti-malarial drugs.

All the patients were given scored tablets of CQ (Lote FCV 002A, Macleods Pharmaceuticals Ltd., India) at a dose of 25 mg/kg, divided into three daily doses: 10 mg/kg on days 1 and 2 and 5 mg/kg on day 3. Patients presenting vomiting half-an-hour after drug administration were given a new treatment cycle with the same dose, and patients with two new vomiting episodes were excluded from the study. Drug administration was strictly supervised by the study personnel, and dosing strategies based on age bands were not used.

The patients included in the study were followed over 28 days with 5 clinical and laboratory evaluations according to WHO guidelines for the assessment of therapeutic efficacy [13]. Data regarding the height, weight and BSA of all the patients were collected at admission. CQ and DCQ concentrations in whole blood were determined using high performance liquid chromatography (HPLC) at days 7 and 28 of follow-up. At day 28 of follow up all the patients were given oral primaquine (PQ) 0.5 mg/kg during 7 days according to the treatment schedule of the malaria program of Bolivia. Patients presenting therapeutic failure or found to have mixed malaria were treated with an alternative schedule and excluded from the study.

## Statistical analysis

The BSA was calculated using the Boyd formula (m^2^) = 0.0003207 × height (cm) 0.3 × weight (g) 0.7285 – [0.0188 × log (weight)] [35], and the dose of CQ in mg/m^2^ according to BSA was calculated using the formula (BSA/1.73) × 1500 (adult dose) [17]. These variables were compared with CQ blood concentrations at day 7 of follow up [36].

The statistical package SPSS® 21 (Chicago, IL, USA) was used to describe and express the variables studied. The normality of the quantitative variables and the residual values of the regression models were evaluated using a QQ-plot. Associations among variables were analysed by comparison of means using the Chi square or Fisher’s exact test and adjusting the linear or curvilinear regressions. Kaplan–Meier analysis was used to estimate the rate of therapeutic failure. Estimations were made using a 95 % confidence interval (CI), and all contrasts of hypothesis were evaluated with an alpha risk of 5 %.

## Results

A total of 96 patients diagnosed with *P.vivax* malaria were administered CQ at 25 mg/kg and CQ + DCQ concentrations in blood were assessed at day 7 of follow up. The details of the therapeutic evaluation have been described elsewhere [33]. One patient was excluded for having atypical height and weight values.

Table 1 shows the characteristics of the patients in each group including: weight, BSA, CQ dose in mg/kg (real dose), CQ dose calculated in mg/m^2^ of BSA (theoretical dose), and CQ + DCQ concentrations in blood on day 7 of follow up (CQD7).

**Table 1:** Characteristics: weight, BSA, CQ real-dose, CQ theoretical dose, and blood concentrations of CQ on day 7 of follow-up in each group

Age group (years)	Number records	Weight (kg)	BSA^a^ (m^2^)	CQ real dose^b^ (mg)	CQ theoretical dose^c^ (m^2^)	CQD7^d^ (ng/ml)
5–9	10	21.15 (SD 5.02)	0.83 (SD 0.13)	536.50 (SD 120.69)	717.71 (SD 112.48)	235.47 (SD 69.92)
10–14	14	42.92 (SD 9.02)	1.33 (SD 0.18)	1085.50 (SD 231.58)	1156.80 (SD 156.32)	308.20 (SD 89.69)
15–24	36	61.42 (SD 9.48)	1.68 (SD 0.15)	1542.81 (SD 239.85)	1458.84 (SD 133.87)	342.72 (SD 165.28)
25–34	14	62.12 (SD 12.60)	1.68 (SD 0.20)	1562.29 (SD 322.06)	1460.28 (SD 174.16)	455.35 (SD 137.86)
35–44	11	71.32 (SD 12.94)	1.83 (SD 0.19)	1782.82 (SD 323.69)	1589.72 (SD 162.42)	395.02 (SD 199.02)
45–54	6	64.67 (SD 8.74)	1.74 (SD 0.13)	1604.08 (SD 238.12)	1510.06 (SD 115.54)	384.57 (SD 51.47)
55–65	4	69.75 (SD 13.60)	1.80 (SD 0.21)	1745.00 (SD 342.08)	1564.96 (SD 183.79)	391.20 (SD 94.90)

*CQ* chloroquine, *DCQ* desethylchloroquine, *SD* standard deviation

^a^BSA in m^2^ = body surface area calculated using the Boyd formula

BSA (m^2^) = 0.0003207 × height (cm) 0.3 × weight (g) 0.7285 – [0.0188 × log (weight)]

^b^CQ real dose: CQ taken in mg/kg/weight

^c^CQ theoretical dose: CQ dose according to: (BSA/1.73)*1500 (adult dose)

^d^CQD7: CQ + DCQ on day 7 of follow-up

**Fig. 1:**
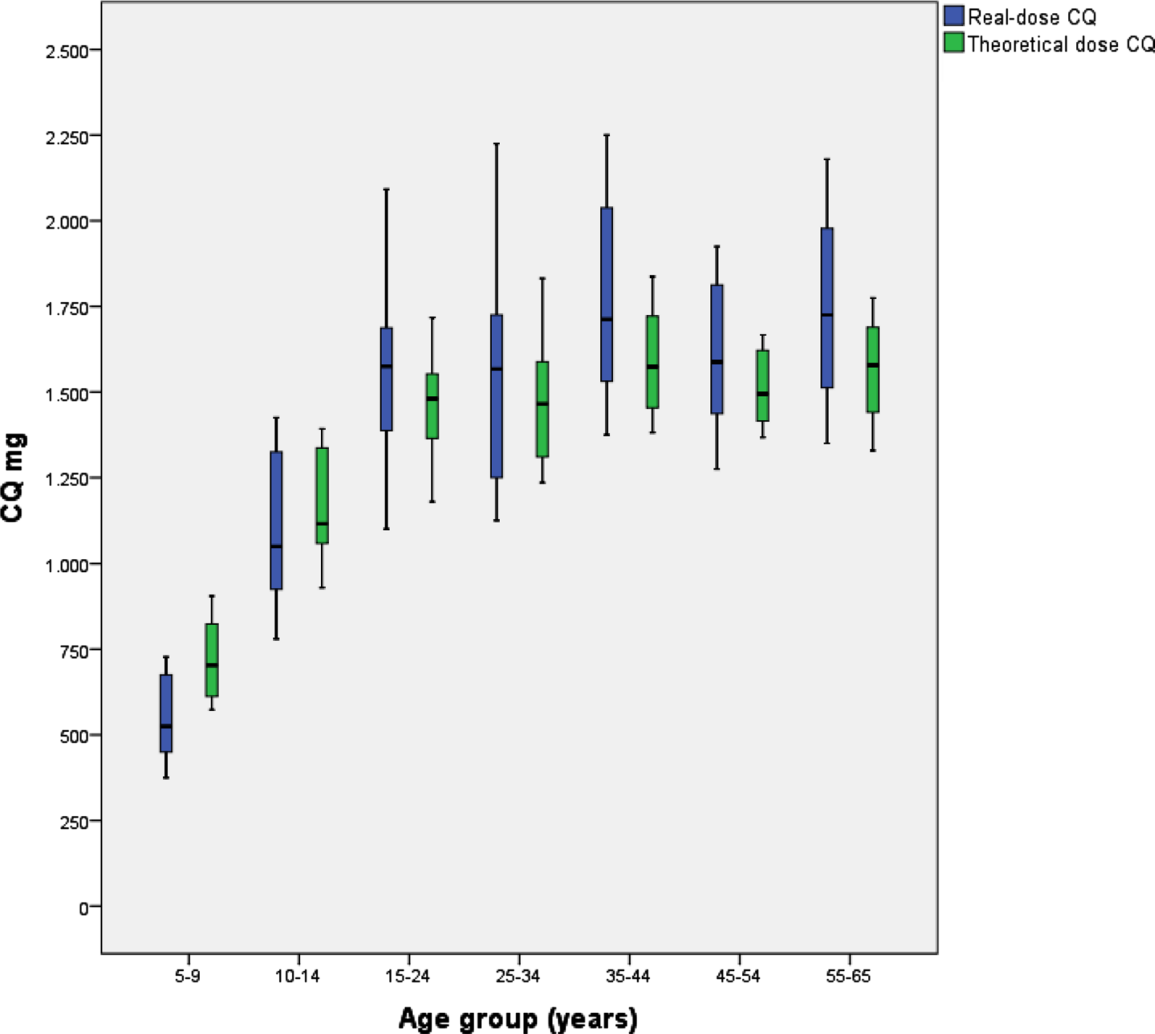
Description of chloroquine (mg) taken in mg/kg (real dose) and dose calculated as mg/m^2^ body surface area (BSA) (theoretical dose) by age group

Figure 1 shows the description of the real and the theoretical CQ dose (mg) by age group. Statistically significant differences were found between the two doses (p = 0.001). In the age groups of 5–9 and 10–14 years the real CQ dose was lower than the theoretical dose, being −181.206 mg (CI 95 % −195.39; −167.02 mg) and −71.30 mg (CI 95 % −118.61; −23.99 mg), respectively.

In contrast, the real CQ dose in patients over 15 years of age was greater than the theoretical CQ dose, with the difference between the two doses being 114.91 mg (CI 95 % 82.64; 147.16 mg).

**Fig. 2:**
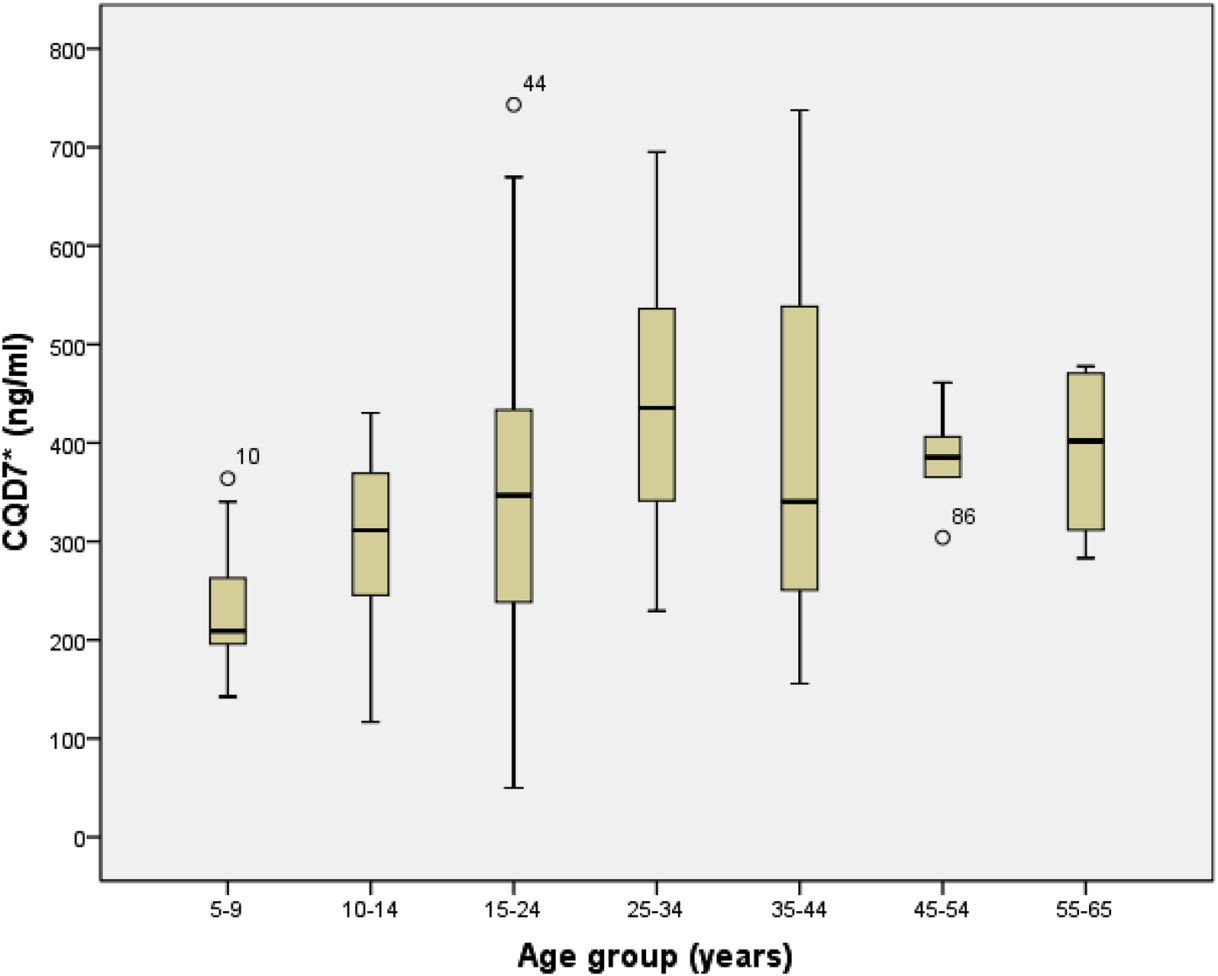
CQD7 (ng/ml) description and analysis by age group. *CQD7 (ng/ml): CQ + DCQ on day 7 of follow up

On analysing the variable “Difference in dose” defined as the difference between the real and theoretical dose, it was found that the younger the study population the larger the difference. Figure 2 shows a logistic model of the relationship of the difference in mg of the two doses and the age (R2 = 0.529, p = 0.001). The difference between the two doses was negative in patients under 15 years of age and could be interpreted as under dosing of CQ compared to the theoretical dose, while the difference was equal to or positive in patients over 15 years.

**Fig. 3:**
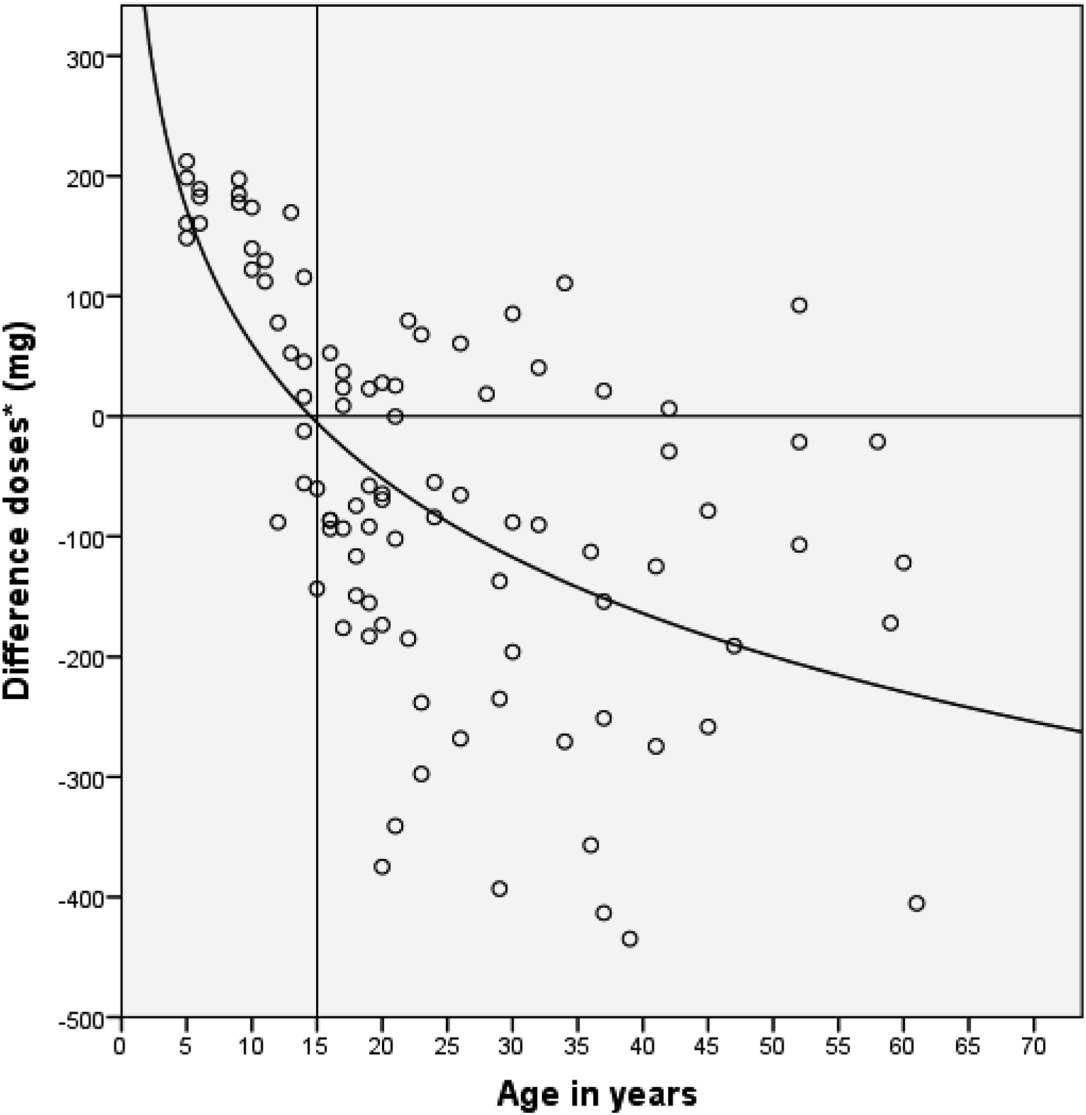
Correlation between the different doses of chloroquine (mg) and patient age (years). *Difference in doses (mg): difference between dose taken in mg/kg/weight and that calculated as mg/m^2^ of body surface area (BSA)

Figure 3 describes the CQ concentrations on day 7 (CQD7) by age group. These concentrations were found to be lower in children under 15 years of age compared to those over this age, with a mean difference of −101.40 ng/ml (p = 0.008). Indeed, the difference was even greater in the group from 5–9 years of age compared to those over the age of 15, being −143.831 ng/ml (p = 0.033).

**Fig. 4:**
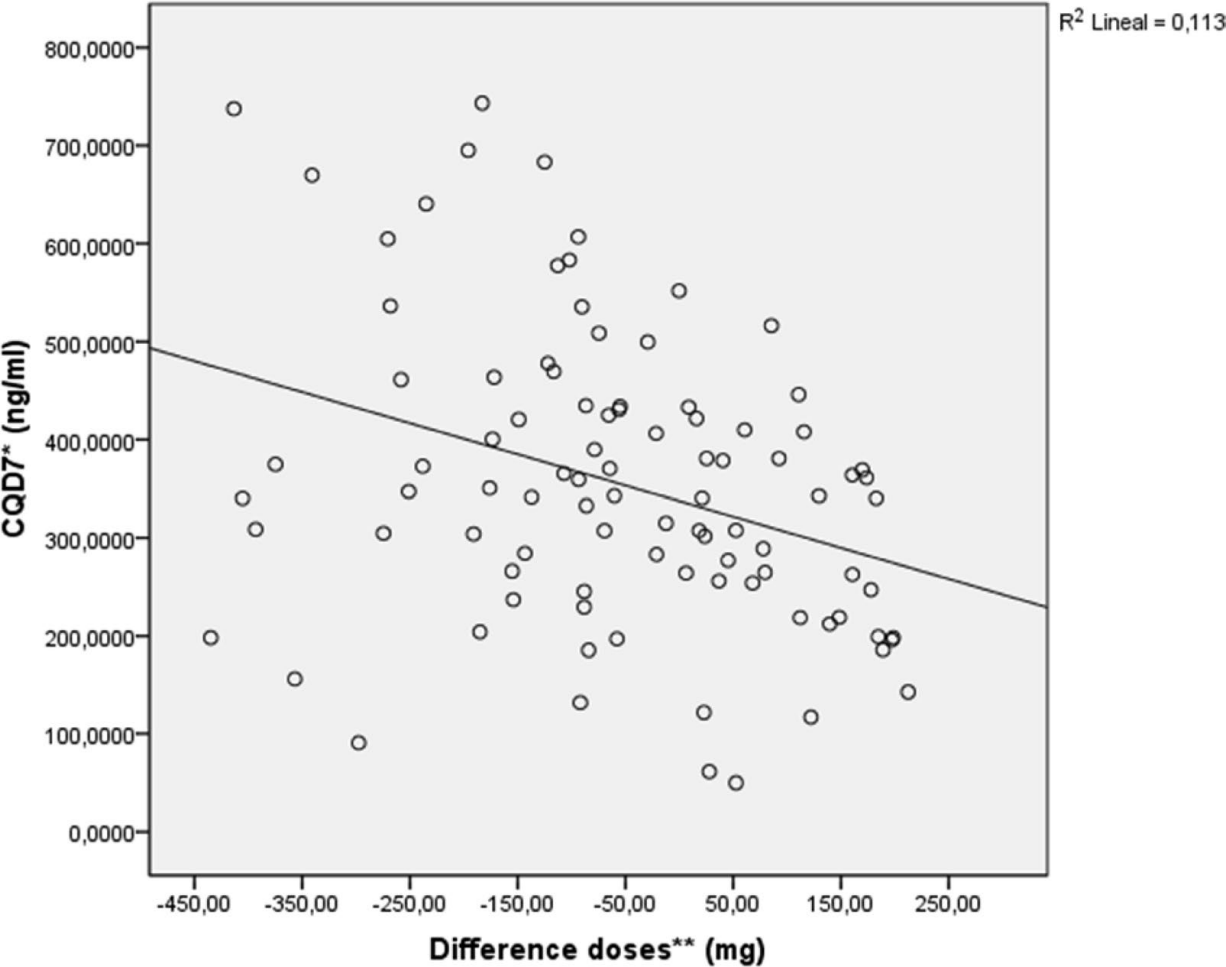
Correlation between the different doses of chloroquine (mg) and chloroquine blood concentrations (ng/ml). *CQD7 (ng/ml): CQ + DCQ on day 7 of follow up. **Difference in doses (mg): difference between dose taken in mg/kg/weight and that calculated as mg/m^2^ of body surface area (BSA)

In relation to the difference between the real and theoretical dose of CQ, it was observed that the lower the real dose the lower the CQD7 values and the greater the difference with the theoretical dose. That is, a negative correlation was found between the difference of the dose (mg) and the CQD7 (ng/ml) (r = −0.337, p = 0.001) (Fig. 4).

On the other hand, of the 95 patients who fulfilled the criteria of normality, nine presented therapeutic failure: 1 on day 7, 2 on day 21 and 6 on day 28 of follow up. The mean age of the patients with therapeutic failure was 14.78 years and that of patients who were cured was 25.20 years (p = 0.03).

On analysing therapeutic failure in relation to age group, the rate of therapeutic failure was found to 28 % in the groups younger than 15 and 4.2 % in the group greater than or equal to 15 years of age (Kaplan–Meier p = 0.005).

## Discussion

The real and theoretical doses of CQ are different and vary with age (p = 0.001). The greatest difference was found in the 5–9 year age group followed by the 10–14-year-old group. On average these groups were administered −181.20 and −71.30 mg, respectively of CQ. To the contrary, patients over 15 years of age are prescribed more than they actually need with 114.91 mg. The relationship between the difference of the two doses and the age was adjusted in a logarithmic model and showed that the younger the age of the individual treated the lower the quantity of CQ prescribed.

With the dose of 25 mg/kg of body weight at diagnosis of *P. vivax* malaria, CQ concentrations in blood on day 7 of follow up were also lower in the youngest age group. The 5–9 years of age group was given an average of 143.831 mg/ml less than patients older than 15 years. These results are similar to those reported by Ringwald et al. [37] who suggested that this could be due to an accelerated elimination of the drug in children.

Using doses of 25 and 50 mg/kg of CQ in children under 15 years of age with *P. falciparum* malaria, Ursing et al. [32] also found that the younger the patients the lower the CQ concentrations at day 7.

Some authors have described the need to take into account that the renal and liver function, metabolic rate, total body and extracellular water, body fat distribution and muscle mass are different in children compared to adults [14, 16, 38]. This could probably explain the differences associated with drug absorption, distribution, metabolism and excretion in children which lead to altered pharmacokinetics and may be the reason for the low CQ concentrations observed on day 7.

Previous studies using allometric scaling models have shown that the cause of the lowest CQ concentrations is probably due to under dosing [39, 40]. In this study it was also observed that the lower the age the greater the difference of the real and theoretical doses. A negative correlation was also found between the real and the theoretical dose of CQ calculated and age, and it was shown that the greater the difference in the dose the lower the CQ concentrations in blood on day 7 of follow up, suggesting under dosing of CQ in children.

The association of the low level of CQ on day 7 found in children under 15 years and the rate of therapeutic failure of 28 % in this same group versus 4.2 % in subjects over 15 years may help to interpret these results as suboptimal dosage of CQ in children under 15 years of age.

These findings suggest the need to consider adjusting the paediatric doses of CQ. Some authors recommend the dose be increased between 35 and 50 mg/kg in children in order to achieve the same CQ concentrations on day 7 of follow up as those of adults [32, 40]. Indeed, several studies have shown that doses of up to 50 mg/kg of CQ can be given to children less than 15 years of age with the same adverse effects as those of a dose of 25 mg/kg [39, 41, 42].

## Conclusion

The standard dose of CQ of 25 mg/kg in the treatment of *P. vivax* malaria may be suboptimal in children under 15 years of age. This under-dosage may be related to a reduction in CQ concentrations found in blood on day 7 of follow up, with younger children showing greater reductions. Indeed, the rate of therapeutic failure in children under 15 is greater than that in patients over this age probably due to this insufficient CQ doses in these patients.

Since suboptimal drug concentrations increase the risk of therapeutic failure and/or drug resistance, paediatric doses of CQ for the treatment of *P. vivax* malaria should be re-evaluated for each age group [23].
